# Beneath the Cedars: Exploring the Water-Energy Balance on Arcellinida Biodiversity in Lebanon’s Cedar Forests

**DOI:** 10.1007/s00248-025-02666-2

**Published:** 2025-12-08

**Authors:** Nura ElKhouri-Vidarte, Fernando Useros, Enrique Lara

**Affiliations:** 1 Department of Mycology, Real Jardín Botánico (RJB-CSIC), Claudio Moyano 1, 28014 Madrid, Spain; 2 Universidad Internacional Menéndez Pelayo, Isaac Peral 23, Madrid, 28040 Spain

**Keywords:** Water-energy balance, Metabarcoding, Microbial diversity, Soil organisms

## Abstract

The distribution of diversity in function of climate has been largely studied in plants and animals, leading to a large body of literature on macroecological rules applied to large geographic scales. However, the applicability of these rules to microbes has almost never been tested at local scales. Arcellinida are a diverse group of protists known to be narrow ecological specialists and constitute therefore an excellent group to test general rules validated on “macrobes”, like the water-energy balance that stipulates that biodiversity peaks with humidity and temperature. In order to test that hypothesis, we collected 122 samples from four cedar forests situated along an elevation gradient in Lebanon, spanning different local climates. We evaluated their diversity using an Arcellinida-specific metabarcoding approach based on the cytochrome oxidase subunit I gene. Our study shows that Arcellinida richness and phylogenetic diversity follow a unimodal distribution, peaking at mid-elevations. β-diversity was chiefly the product of turnover, illustrating the high spatial heterogeneity of the forests. Precipitation and actual evapotranspiration were identified as key drivers of diversity, thus supporting the water-energy balance hypothesis. Communities situated at higher or lower elevation were, to a large extent, subsets of more diverse mid-elevation assemblages, which designates the latter as biodiversity sources. These results suggest that, under the increasing aridification of the Middle East due to climate change, Arcellinida communities will lose diversity and will undergo a process of homogenisation, with possible consequences on ecosystem functioning.

**Supplementary Information:**

The online version contains supplementary material available at 10.1007/s00248-025-02666-2.

## Introduction

Macroecology, as defined and named by Brown and Maurer [1], studies the changes in diversity across time or space. It is a rapidly evolving field that brought a most useful theoretical frame to address the effects of disturbance on species diversity [2–5]. Macroecology researches have been mostly focused on large and multicellular organisms (i.e. macroorganisms or macrobes) while similar studies on small and unicellular organisms (i.e. microorganisms or microbes) are still in their infancy [6]. However, quoting Shade et al. [6] there is a common goal in understanding the causes and consequences of changes in biodiversity, and therefore macroecology should not be detached from microbial ecology. The large scales studied in macroecology influence also diversity patterns at local scales [7, 8]. Indeed, more than ever, we need a holistic view on ecosystems that includes microbes as their most diverse fraction, especially in the actual context of biodiversity loss and climate change [5, 9]. Protists are extremely diverse and constitute the bulk of microbial diversity [10, 11]. Their fundamental role in shaping microbial communities through their predatory action, in carbon fixation and in nutrient cycling (among others) has been widely demonstrated [12–14]. In turn, soil protist diversity has been shown to influence plant resistance to drought and parasites [15], and is therefore important for plant survival under a global change scenario [16]. The relevance of protist diversity has motivated researchers to study their potential drivers at the landscape scale and beyond [13]. Previous research found that topographic and climatic variables had a stronger influence on diversity distribution than local soil parameters [17]. Precipitations seem to be a relevant diversity driver [18], a logical conclusion given the fact that low water availability is an efficient ecological filter for protists [19, 20].

The water-energy balance is one of the most frequently tested macroecological theories to explain the distribution of organisms diversity [21]. This hypothesis postulates that water and energy availability are fundamental drivers of biodiversity patterns, modulating species richness and productivity, showing how species thrive in warm and humid latitudes [22, 23]. Similarly, elevation gradients provide diverse climatic areas over shorter distances and present a known pattern in which temperature decreases with elevation, while precipitation shows a more complex, non-linear pattern [24, 25]. However, while this model has shown to be very powerful to explain diversity distribution in plants and animals, microorganisms remained practically unstudied [26]. Surveys of microbial diversity at the landscape scale were technically impossible until relatively recently, but the wider accessibility of metabarcoding has been a big breakthrough in eukaryotic environmental microbiology [27, 28], allowing large surveys of hundreds of samples that are needed to identify macroecological patterns at a lower cost than previous tools [6, 29]. The use of variable markers, such as the first subunit of the mitochondrial cytochrome oxidase gene (COI), also proposed as an universal marker of the Barcode of Life [30], allows a taxonomic precision that can be used as a proxy for species discrimination reaching the species level and beyond [29]. This level of accuracy is needed to allow comparisons with studies on “macrobes”, but requires protocols specifically adapted to single taxa [31].

Arcellinida is a diverse clade of protists, ubiquitous in terrestrial systems. This group of testate amoebae includes relatively large species (most often > 50 μm) and are considered among the top predators in the microbial world [32, 33]. As such, they prey on a variety of bacteria, fungi and other small eukaryotes [32], and depend thus on balanced foodwebs to thrive. They have often restricted ecological niches, and distinct communities can be found associated with microbial mats in karstic caves [34, 35] or to particular microniches in boreal peatlands [36]. Because of these characteristics, Arcellinida diversity can be linked to ecosystem functioning, a hypothesis that is corroborated by their use as bioindicators of soil health [37, 38]. Current knowledge on the effect of temperature and precipitations (in line with the water-energy balance model) on Arcellinida diversity appears nevertheless contradictory; Arcellinida diversity peaked under wetter and warmer climates in Chile [22]; conversely, another study shows evidence for an increase in diversity under colder climates in Russia [39]. When considering elevation gradients, one study in the Swiss Alps showed a decrease in diversity in lower altitudes [40]; this result is contrasted with another gradient in the Alps where diversity peaks at higher altitudes [39]. These contradictory results illustrate that, while climate undoubtedly has an effect on Arcellinida diversity, other confounding drivers probably play also a major role. For instance, the differences in soil characteristics and sampling design between the studies are probably affecting the results [41]. Moreover, the type of vegetation alone has been shown to explain more than 10% of the variation in testate amoebae communities [41]. Therefore, in order to test the water-energy balance on soil microbes, it is necessary to reduce as much as possible confounding factors, such as different substrates or associated plants.

In that purpose, we sampled litter as a habitat for Arcellinida in four cedar forests (*Cedrus libani*) situated at different elevations in Mount Lebanon. All these forests are located within Natural Reserves, which limits as much as possible heterogeneity resulting from human impact. Mount Lebanon is a karstic mountain range, thus buffering the soil to high pH and conductivity [42]. Within the sampled forests, litter is almost exclusively composed of *C. libani* needles, which removes variability associated to different vegetation types. By sampling exclusively *C. libani* litter within protected cedar forests that grow in karstic landscapes, we reduced as much as possible the confounding factors that might have been influencing results in earlier studies. In order to measure diversity with a high taxonomic accuracy, we applied a recently developed Arcellinida-specific metabarcoding protocol based on the mitochondrial marker gene cytochrome oxidase (COI), ensuring species-level discrimination [29]. In accordance with the water-energy balance model, we predict that richness will peak at sites where both humidity and temperature are highest. Furthermore, we predict that the less favourable sites, meaning the ones with less water-energy balance, will host a subset of the richest sites, as Arcellinida diversity has been found to be driven by ecological filters rather than interspecific competition [36]. Finally, we will discuss the consequences of global change on Arcellinida diversity and, by extension, on the soil microbiomes associated to the Lebanese cedars.

## Material and Methods

### Area of Study

**Fig. 1:**
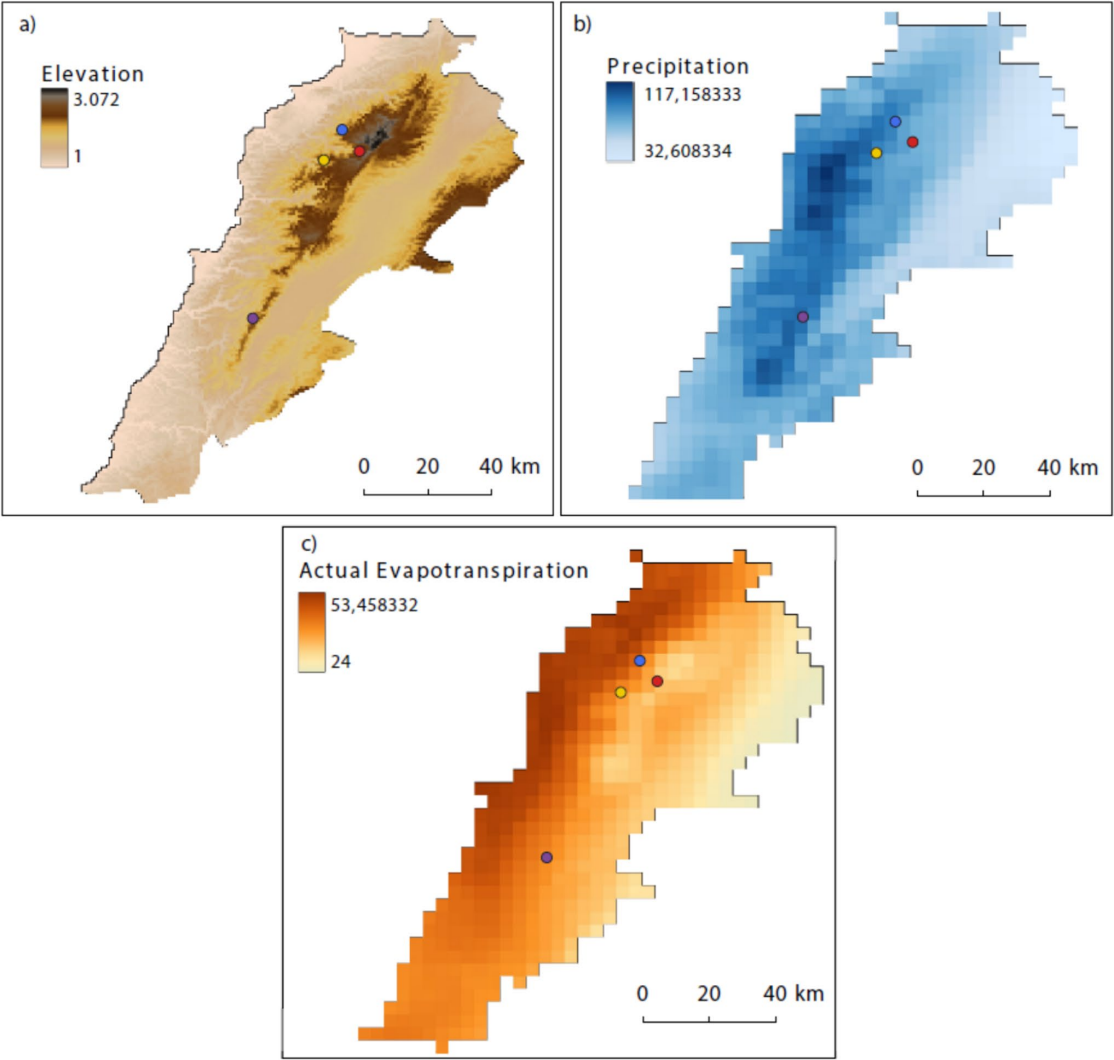
Maps of Lebanon showing the four study sites: Shouf in purple, Tannourine in yellow, Ehden in blue and Bsharre in red. The map **a**) represents the elevation in the country with a spatial resolution of 30 s (~ 1km^2^, WorldClim, [50]), **b**) the mean annual precipitation between 2020 and 2021 with a spatial resolution of 2.5 min (~ 21km^2^, Climatology Lab - Home, [49]) and **c**) the actual evapotranspiration (AET) from 1991 to 2020, with a spatial resolution of 2.5 min (~ 21km^2^, Climatology Lab - Home, [49])

Lebanon is a small country (10,452 km^2^) with a high climatic diversity given the different biomes created by its high mountains, valleys, rivers and the Mediterranean Sea. Lebanon, as a country, is therefore well suited for climatological assessments and modelling researches [43]. It harbours a large diversity of endangered fauna and flora and counts with several Protected Areas, most of them protecting the Lebanese Cedar (*Cedrus libani*), a Middle East endemism [44]. However, not much is known about the microbes diversity in Lebanon [45]. Cedar forests in Lebanon have been exploited by humans for the last 5000 years, and while they once covered up to 70% of the country, they are nowadays severely fragmented and have decreased to 13% [46]. While *C. libani* forests represent less than 1% of the total forest cover in Lebanon, they remained under strict protection, some of them for at least 30 years [47, 48]. As study sites, we chose four of the relic forest patches of Lebanese cedars located across the western slope of the Mount Lebanon chain and with altitudes ranging from 1500 m to almost 1950 m: Shouf, Tannourine, Ehden and Bsharre. The characteristics of each sampling site are provided in Table 1. A detailed description of the sampling sites is provided in the Supplementary Information Appendix 1 (Fig. 1).

**Table 1:** Mean elevation, precipitation, maximum temperature, minimum temperature and actual evapotranspiration for each sampling site

Site	Mean elevation (m)	Average annual precipitations (mm)	Average annual max_temperature (ºC)	Average annual min_temperature (ºC)	Average annual AET (mm)
Ehden	1585	1073	19.2	7.8	528
Shouf	1716	1185	20.7	9.1	491
Tannourine	1770	1095	18.6	6.8	485
Bsharre	1929	926	15.6	3.5	415

The data have been obtained from rasters layers. The precipitation layer had data between 2020 and 2021 with a spatial resolution of 2.5 min (~ 21km^2^, Climatology Lab - Home, [49]), the actual evapotranspiration (AET) from 1991 to 2020, with a spatial resolution of 2.5 min (~ 21km^2^, Climatology Lab - Home, [49]), the elevation had a spatial resolution of 30 s (~ 1km^2^, WorldClim, [50]) and the minimum temperature, maximum temperature and the mean annual precipitation had a resolution of 2.5 min (~ 21km^2^, WorldClim, [50])

### Sampling

We collected between 30 and 31 samples per forest, establishing a sampling time of two hours in each to balance the sampling effort. We selected as sampling sites random areas during the two hours walks, always sampling close to the paths and in accessible parts. While we acknowledge that such a design might have introduced biases, the potential presence of unexploded landmines prevented us from performing a fully randomized sampling across the whole forest in certain areas, as for others we followed the regulations of the forests. For each sample, we softly removed the cedar needles and took a handful of soil from the H_0_ horizon, the soil surface (0–5 cm), where we find organic matter in decomposition. The soil was then sieved with a 500 µm mesh sieve with tap water to a Falcon® tube. We collected 1 ml of the precipitated soil with a disposable sterile plastic Pasteur pipette and we transferred it into a 2 ml Eppendorf tube containing 1 ml of LifeGuard™ Soil Preservation Solution (Qiagen).

### eDNA Extraction and Amplification

For the environmental DNA (eDNA) extraction, we used a Qiagen DNeasy PowerSoil Pro Kit following the instructions provided by the manufacturer. The extracted eDNA was conserved at −20 °C. The Arcellinida-specific protocol for eDNA amplification was achieved following González-Miguéns et al. [29], which can be seen in detail in dx.doi.org/10.17504/protocols.io.yxmvm2389g3p/v1. We used readily indexed primers to be able to multiplex the PCR products in the sequencing reaction (Supplementary Information Table 2). The final fragment was 407 bp long. Every successfully amplified sample was purified by band excision and then quantified with a Qubit 3 Fluorometer with dsDNA High Sensitivity (HS) assay kits (ThermoFisher). The samples were sequenced using NextSeq (Illumina) at the Fundación Parque Científico of Madrid, who also purified the products and created the library by adapter ligation. The raw data obtained can be found in Supplementary Information Appendix 2 (Zenodo, 10.5281/zenodo.15363229).

**Table 2:** Results of the generalised linear mixed model with a Poisson distribution, showing the effect of environmental variables on species richness, with the site included as random factor

	Chisq	Df	Pr (> Chisq)
AET	2.6019	1	0.1067
Precipitation	31.7416	1	1.761e-08 ***
NDWI	2.1838	1	0.1395
HH	0.7854	1	0.3755

The table shows the test statistic (Chisq), the degrees of freedom (Df), and the associated *p*-value (Pr(> Chisq)) for each predictor. Significance levels are indicated by stars: *** *p* < 0.001, ** *p* < 0.01, * *p* < 0.05, *p* < 0.1

### Bioinformatics

1. *Data Curation*

The sequencing output was processed using the pipeline described in González-Miguéns et al. [29]. First, Cutadapt v2.8 [51] was used for trimming the primers and demultiplexing. Quality profiles were first assessed using FASTQC v. 0.11.9 [52] and then the R package "dada2" [53], which was also used to filter and trim the sequences, truncating forward and reverse reads after position 260. Paired-end reads were merged in DADA2 to generate amplicon sequence variants (ASVs), and chimeric sequences were removed. The information of the filtered sequences is shown in Supplementary Information Table 3. Taxonomic classification of the ASVs was conducted using VSEARCH v.2.21.1 [54] against a curated database for eukaryotic COI to enhance the performance of the taxonomic assignment [55].

**Table 3:** Results of the generalised linear mixed model with a Gaussian distribution, showing the effect of environmental variables on phylogenetic diversity, with the site included as random factor

	Chisq	Df	Pr (> Chisq)
AET	0.6014	1	0.4380
Precipitation	29.3926	1	5.91e-08 ***
NDWI	0.0051	1	0.9433
HH	1.7629	1	0.1843

The table shows the test statistic (Chisq), the degrees of freedom (Df), and the associated *p*-value (Pr(> Chisq)) for each predictor. Significance levels are indicated by stars: *** *p* < 0.001, ** *p* < 0.01, * *p* < 0.05, *p* < 0.1

**Fig. 2:**
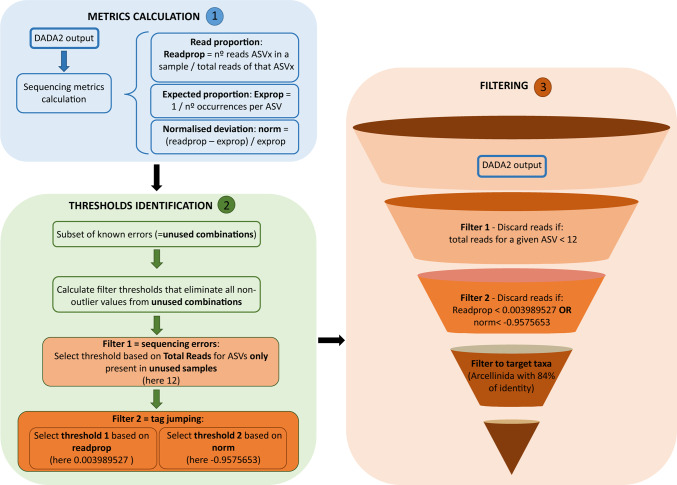
Flowchart describing the steps followed to remove the tag-jumping

We then performed a filtering step to remove possible tag-jumping. Tag-jumping, also known as index hopping, occurs when barcodes (tags) used to multiplex samples are incorrectly transferred between DNA fragments, leading to cross-contamination between samples. This results in the generation of false positives, which blur diversity patterns [56, 57]. In order to reduce their impact as much as possible, we applied a stringent threshold-based filtering to improve result reliability (Fig. 2).

Our approach to the establishment of thresholds is based on the observation of “blank samples” [58], which are index combinations that have not been used in the study. In theory, these “blank samples” should have no reads, therefore any observation including these indices can be considered as known errors. The unused indexed primers combinations are listed in the Supplementary Information Table 3. First, we determined for each observation (an ASV in a sample) the proportion those reads represent with respect to the total number of reads of that ASV (“readprop” in the script). If readprop was 1, that ASV is only found in that one sample. Briefly, we applied three filters, which will be based only on the observations in “blank samples” (known errors).

(i) First we defined the filter 1 (“sequencing errors”) threshold. We considered here all ASVs present only in “blank samples” whose readprop = 1 (unique). These must come from other errors beyond tag-jumping, because they are not found in other samples. We considered the number of reads of these erroneous observations, identifying values above the interquartile range (from now on, “outliers”) using the function “boxplot.stats” of the “grDevices” package [59]. After removing the outliers, we selected as threshold the maximum value (12 reads). Considering the maximum value as threshold, including all the outliers, would have eliminated too many observations and generated a lot of false negatives. This threshold would eliminate the great majority of errors of these type. All ASV occurrences below that threshold will be eliminated.
(ii) Highly abundant ASVs have a higher probability of generating tag-jumping biases [56]. These erroneous observations may have a lot of reads in certain cases, but they should represent a low proportion of the total number of reads of a given ASV. For the filter 2 (“tag jump filter”), after applying the first filter to the “blank samples”, we determined the outliers using the function “boxplot.stats” for the readprop column. We select the minimum value as threshold (0.003989527).
(iii) However, we cannot consider just the readprop, because an ASV that is naturally present in all samples would have lower readprop values. Therefore, we considered the number of samples were an ASV was present and calculate the expected proportion, if it were equally distributed:
  $$exprop=\frac{1}{\text{number of observations of that ASV}}$$
(iv) We then calculated the divergence between the observed proportion (readprop) and the expected (exprop), normalising it by the exprop value. We called that value norm:
  $$norm=\frac{ readprop - exprop }{exprop}$$
  We considered the “norm” value of observations in “blank samples” to establish another threshold for the filter 2, considering the minimum value among the outliers as threshold (−0.9575653).

Once all thresholds were set, we applied the “sequencing errors” filter to non-blank samples. Afterwards, both of the thresholds of the “Tag jump” filters were applied conditionally, meaning that an observation is to be maintained if it passes at least one of the thresholds (readprop > 0.003989527 or norm > −0.9575653). While other studies apply a fixed and arbitrary threshold to eliminate artefactual data in metabarcoding studies, our protocol aims at adapting data filtering to each sequencing experiment in an objective way. The script is available in Supplementary Information Appendix 3.

Finally, we filtered out those ASVs that had an identity value for Arcellinida lower than 84%. This threshold was determined empirically, checking the sequences in GenBank to verify their taxonomic assignation, and considering the COI pairwise distance of the Arcellinida order [29, 55].

2. *Tree, Clustering and Analyses*

After the data curation, we aligned all our sequences with those of the database for eukaryotic COI [55], also including all the barcoded species known for Arcellinida as well as the ASVs from previous metabarcoding studies that used the same eDNA extraction and amplification protocol [29, 34, 60, 61] using MAFFT [62] as implemented in Geneious Prime Bioinformatics Software v2.1.2023 [63]. Afterwards, a phylogenetic tree was constructed with the ASVs. A maximum likelihood (ML) tree was generated using IQ-TREE v1.6.12 [64] with a General Time Reversible (GTR) model, and node support was assessed with 1000 bootstrap replicates. The resulting trees were refined using FigTree v1.4.4 [65] and R v4.3.2 [59].

Using a distance matrix obtained with Geneious Prime with the ASV tree as input, ASVs were then grouped into Operational Taxonomic Units (OTUs). We determined these OTUs as approximations of species, based on a barcoding gap of 3% as determined in [34, 58, 66, 67] and earlier publications [68]. Genetic distances between ASVs were calculated using a distance matrix in Geneious Prime. Clustering was performed in R v4.3.2 [59] with the “DECIPHER” package [69], using the “complete method”, meaning that the maximum distance between any pair of ASVs from a cluster is 0.03. From the ASV tree, we used the “ape” package [70] to transform the ASV tree in an OTU tree, leaving only one representative per OTU. To visualise the clades and the distribution of OTUs among samples, we used the R packages “ggtree” [71] and “phytools” [72].

To obtain data from climatic variables, we downloaded the layers from the Climatology Lab, WorldClim and Google Engine [49, 50, 73]. We then extracted the data for our coordinates with the package “raster” [74], and we resampled the rasters to normalise their resolution to the higher one with the function resample() of the package “raster” [74] (Supplementary Information Appendix 3). Precipitations and temperature were highly correlated in our sampling sites (> 99%), hence we removed temperature from the selected variables. We rather used actual evapotranspiration (AET), which has been shown to be highly correlated with net primary productivity, microbial respiration, and species richness, particularly in dry or seasonal environments [75]. Other variables associated to the water-energy balance paradigm are the horizontal polarisation of the PALSAR radar(HH), which assesses soil humidity, and the normalised difference water index (NDWI) (Supplementary Information Table 1). Finally, we assessed the variance inflation factor (VIF) for the selected variables using the package “car” [76] (Supplementary Information Table 4).

To analyse the data and their correlations with the environmental variables, we used the R package “phyloseq” [77] as it allows to create an object with the phylogenetic tree and the abundance matrix incorporated. To obtain the graphs we used “ggplot2” [78] and “VennDiagram” [79]. To calculate the richness, in terms of number of OTUs, we used the function alpha() from the “microbiome” package [80], and to calculate the phylogenetic diversity (PD) we used the function pd() from the “picante” package [81] with the OTUs tree. The phylogenetic diversity refers to the total sum of the phylogenetic distance among species present in a given community [82]. The pd() function calculates Faith’s phylogenetic diversity, by calculating the sum of the total phylogenetic branch length. Prior to the analyses, we calculated the residuals of the lineal model between OTU diversity and sequencing depth, which represent the residuals variances in richness, not explained by the sequencing depth [83, 84]. This was done in order to standardise and remove the sequencing depth bias, and therefore the residuals can be interpreted as 'depth-corrected diversity' values. A positive residual means the sample has more richness than what it was predicted in the model [58]. The statistical analyses of the alpha diversity were calculated using the adonis2() function from the “vegan” package [85]. In order to characterise the beta diversity within each protected area, we partitioned the beta diversity into its two independent components (nestedness and turnover). Turnover is defined as the replacement of species between sites due to ecological filtering or competition, while the nestedness is a pattern where diversity in some sites is a subset of other sites, implying gain or loss without replacement, due, for example, to dispersal limitations [86, 87]. We assess the nestedness and turnover using the package “betapart” [88]. To visualise the NMDS we used the function metaMDS() from the package “phyloseq” [77].

The scripts used to obtain these results can be found in Supplementary Information Appendix 3.

## Results

In total, 35,791,846 quality filtered reads from 38,233 ASVs were recovered from the 122 samples amplified and 10,366,813 were discarded from further analyses with DADA2 for being chimeras or singletons. After a second filtration to remove potential tag-jumpings, and non Arcellinida sequences (below 84% identity with Arcellinida), we retained 13,833,562 reads from 3,330 ASVs. After clustering these ASV into OTUs we obtained a total of 697 OTUs (following a 3% threshold).

**Fig. 3:**
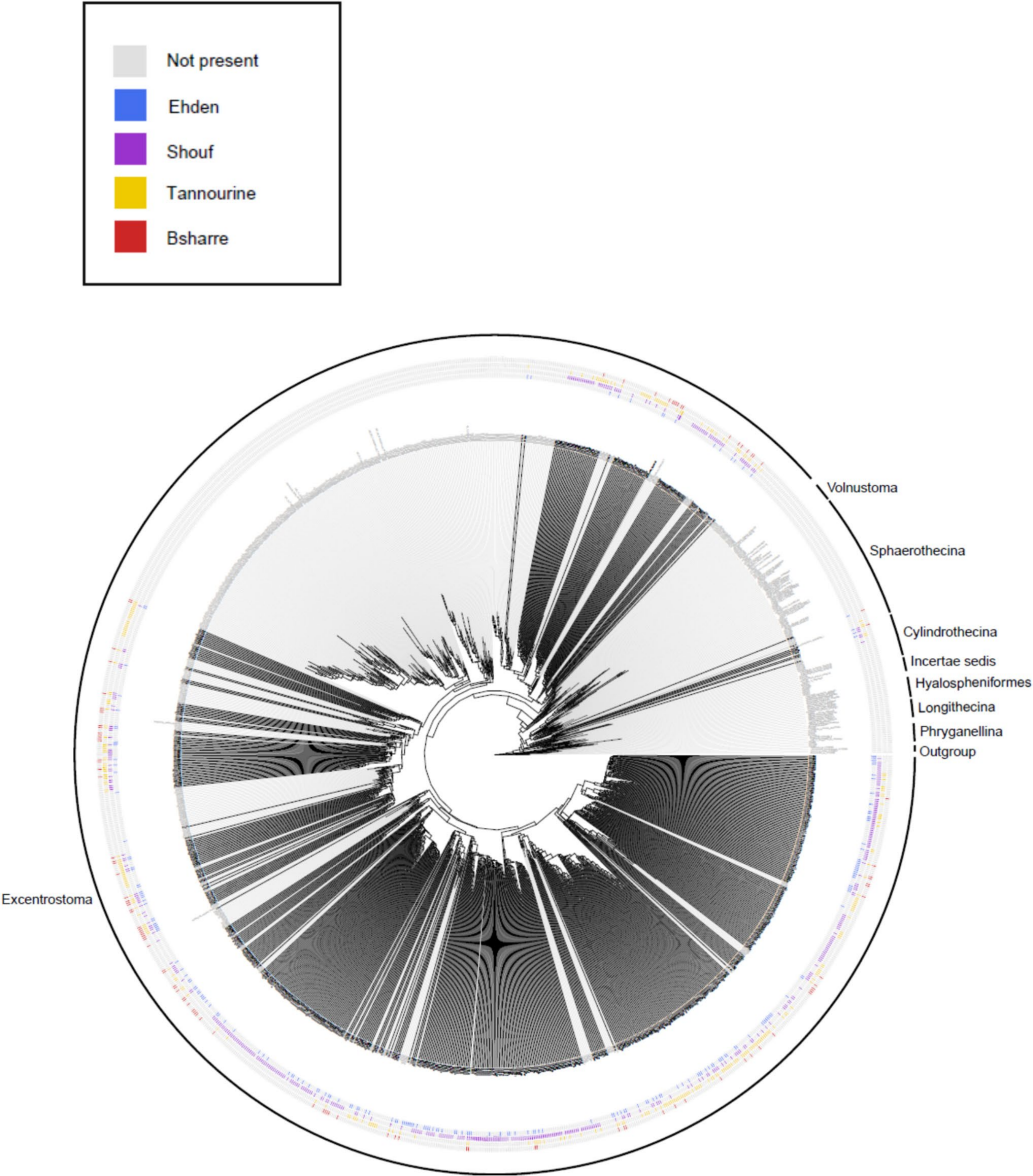
Maximum likelihood phylogenetic tree of Arcellinida based on a fragment of COI. The black branches represent the OTUs containing at least one sequence obtained in this study, while the grey braches represent OTU including solely sequences from the database. The branches with names are OTU in which we can find a sequence obtained with barcoding, for which we have the species or at least the genus. The outgroup was formed by four species from the Euamoebida orders closely related to Arcellinida: *Copromyxa* sp. (LC102283), *Copromyxa protea* (LC102284), *Copromyxa cantabrigiensis* (LC102285) and *Saccamoeba* sp. (LC102286)

We mostly obtained sequences of infraorder Excentrostoma (688 OTUs and 13,414,410 reads), and a few sequences of Cylindrothecina (7 OTUs and 412,015 reads), and Sphaerothecina (2 OTUs and 7,137 reads, Supplementary Information Table 5). Figure 3 indicates the phylogenetic relation between our OTUs and sequences from other studies, indicating also in which parks they were found. The number of Illumina reads per sample is detailed in Supplementary Information Table as well as the number of reads assigned to Arcellinida.

**Fig. 4:**
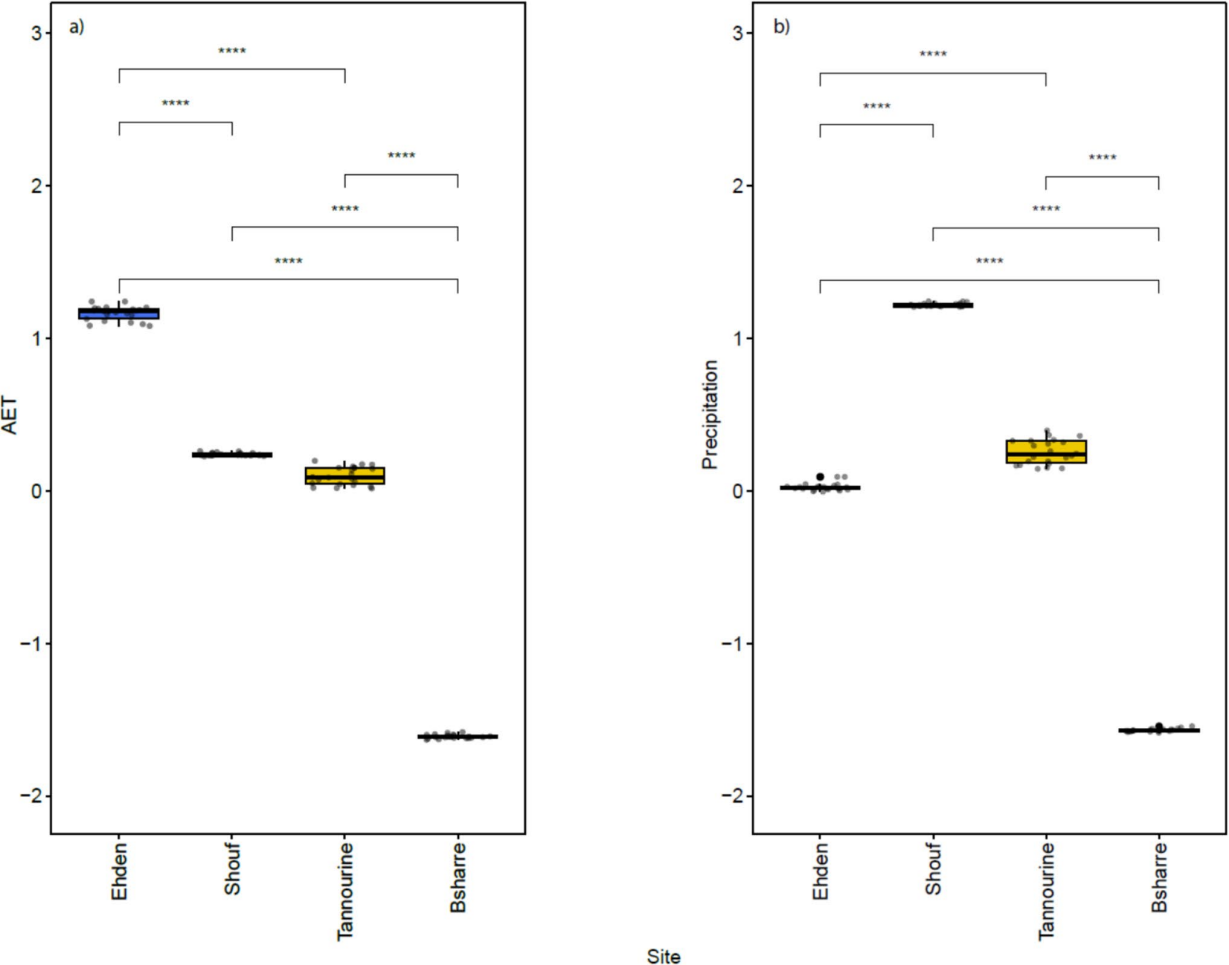
Boxplots showing **a**) the AET and its difference between sampling sites and **b**) the precipitation and its difference between sampling sites. The significance was calculated with the Wilcoxon test. The symbol * represents *p* < 0.05, ** *p* < 0.01, *** *p* < 0.001 and **** *p* < 0.0001

Regarding the sampling sites, the climatic parameters varied between protected areas. The AET and precipitation showed statistically significant differences among the study sites. Ehden showed the highest value of AET, followed by Shouf. The highest precipitation was found in Shouf, followed by Tannourine. Bsharre had the lowest values for both precipitation and AET (Fig. 4).

**Fig. 5:**
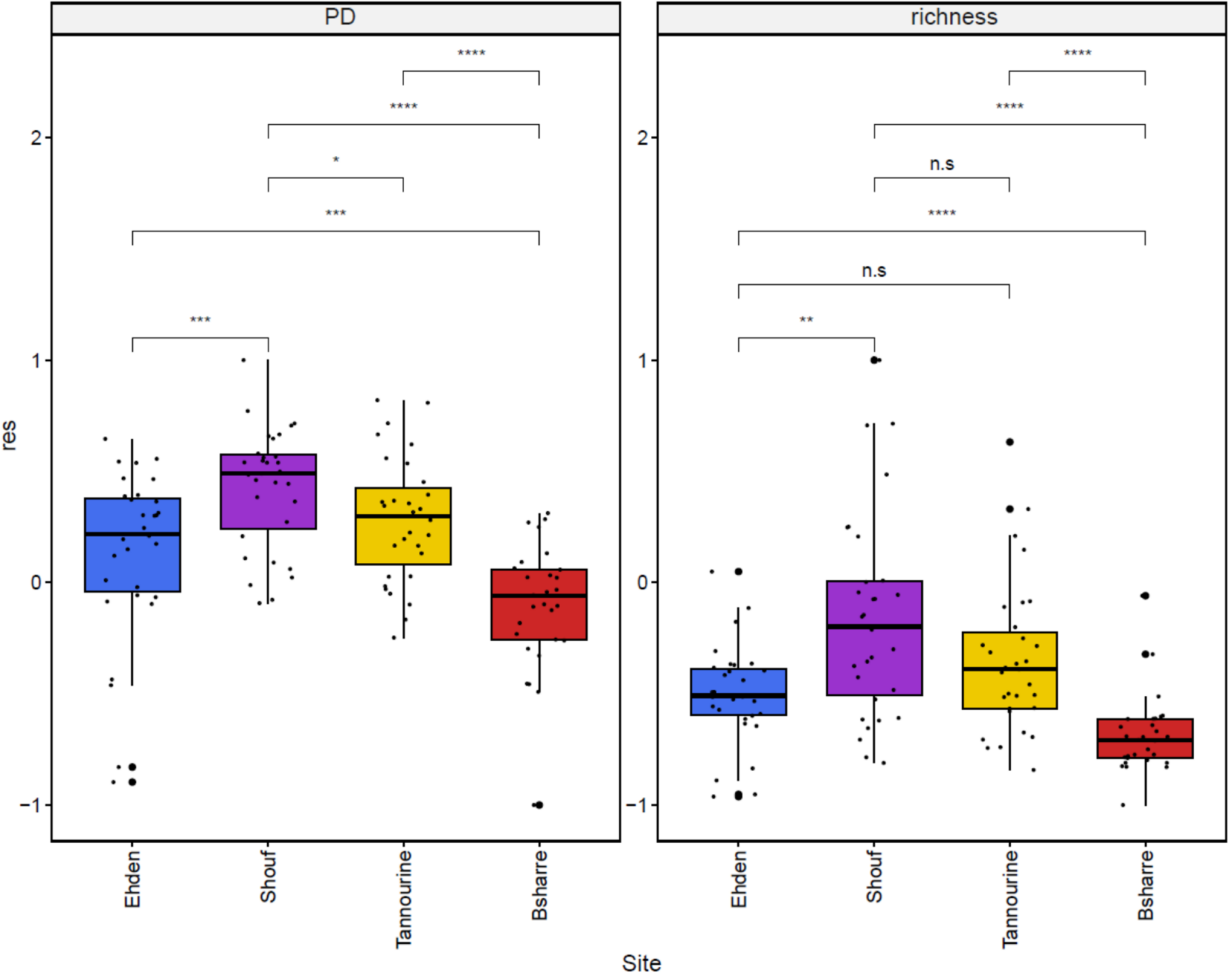
Boxplots representing the scaled residuals of two alpha diversity indexes after accounting for the effect of sequence depth in each sampling site. Phylogenetic diversity (PD) is shown on the left and richness on the right. The significant differences between sites were evaluated using the Wilcoxon test and are denoted as follows: **** (*p* < 0.0001), *** (*p* < 0.001), ** (*p* < 0.01), * (*p* < 0.05), n.s (not significant)

**Fig. 6:**
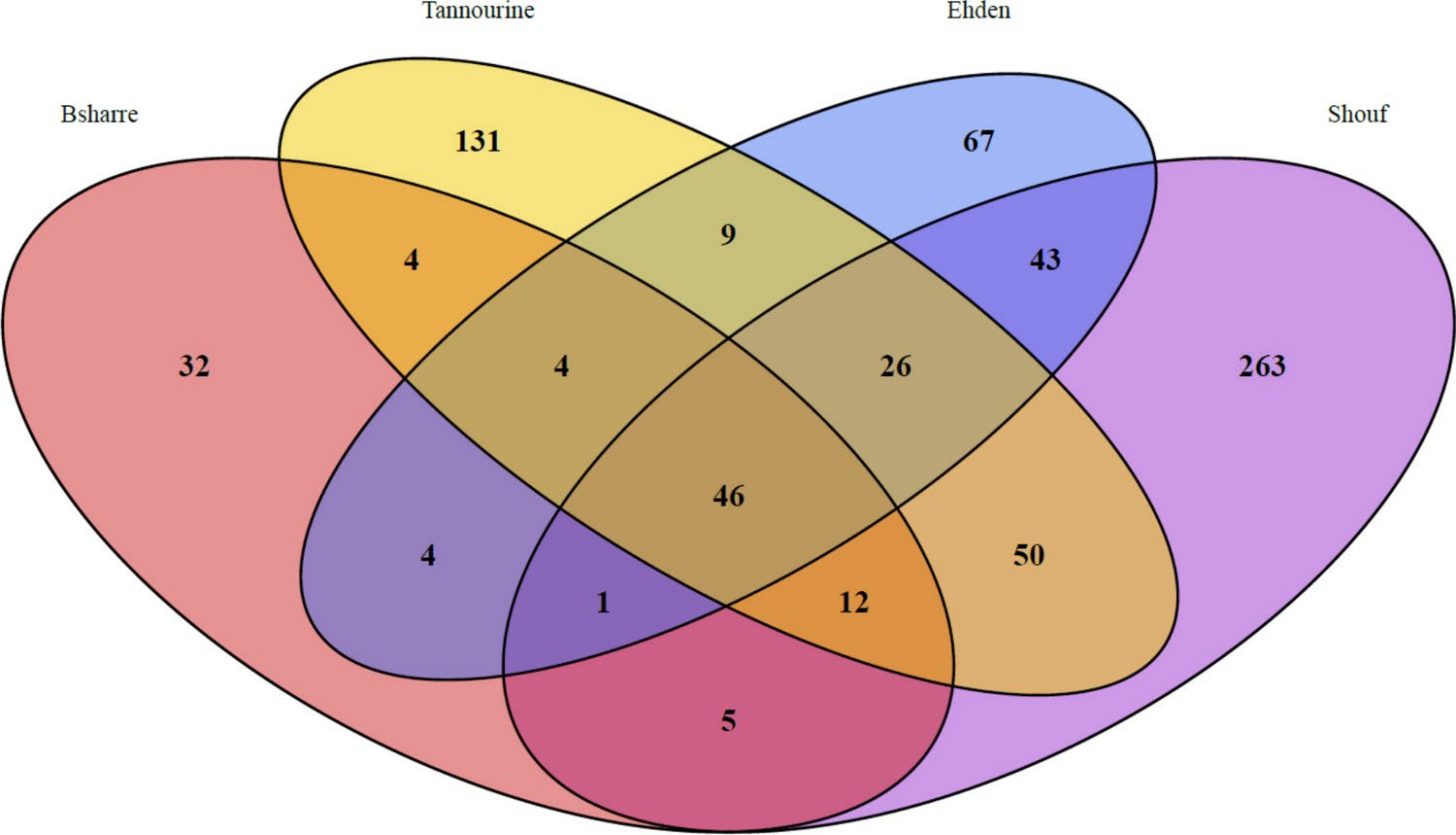
Venn diagram showing the number of OTUs present in each one of the sampling sites. Purple represents Shouf, blue represents Ehden, yellow represents Tannourine and red represent Bsharre

We noticed significant differences in richness (alpha diversity) between protected areas. Shouf has the highest values for both richness and phylogenetic diversity (PD), followed by Tannourine and Ehden, although the difference in richness between Shouf and Tannourine is not significant (Fig. 5). The difference between Ehden and Tannourine in both richness and PD was not significant (Fig. 5). Bsharre had a statistically significant lower richness and PD than the rest of the protected areas. Shouf was the protected area that had most exclusive OTUs (not shared with the other sites; 263 exclusive OTUs) followed by Tannourine (131 exclusive OTUs), Ehden (67 exclusive OTUs), and Bsharre (32 exclusive OTUs; Fig. 6).

We checked the effect of the climatic variables with a generalised linear mixed model, including the site as a random effect. We checked for overdispersion, and reduced the models. Precipitation was found to have a statistically significant effect on both the species richness and thephylogenetic diversity (Tables 2 and 3).

**Fig. 7:**
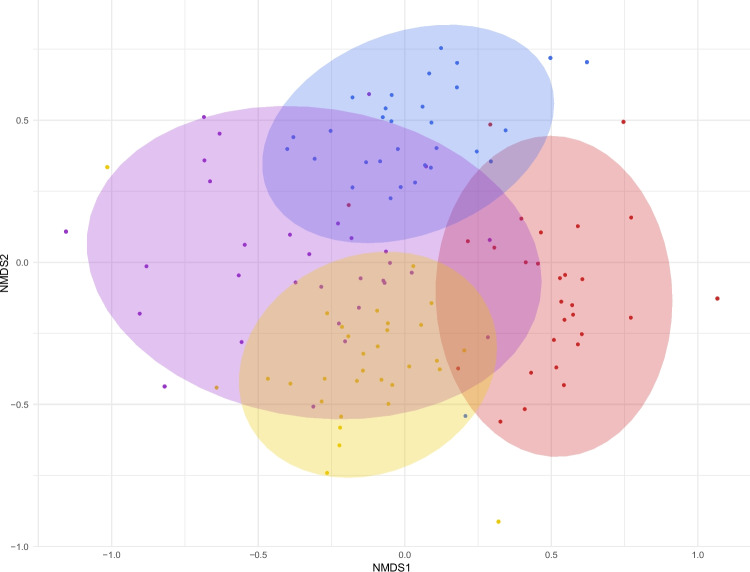
NMDS ordination plot based on Jaccard dissimilarity matrix for the presence and absence of the OTUs in each of the sampling sites. We chose k = 3 and the stress was 0.20

An NMDS analysis showed marked differences between the communities found in the different protected areas (Figs. 3 and 7), although there are overlaps, especially between Shouf and Tannourine, and between Shouf and Ehden. Tannourine and Ehden did not overlap. Bsharre showed an outstretched shape and overlapped with all the other sampling sites (Fig. 7). The stress curve of the NMDS can be accessed in Supplementary Information Appendix 4.

The PERMANOVA analysis showed that the precipitation was the environmental variable which was most strongly correlated with Arcellinida community diversity (5.4%), followed by the actual evapotranspiration (AET, 4.0%). The HH and NDWI had a lower contribution (1.2% and 1% respectively) but were still statistically significant (Table 4).

**Table 4:** Results of the PERMANOVA analysis based on a Jaccard distance matrix, performed using the adonis2 function in R with four climatic variables as predictors

Variable	R^2^	*P*-value
AET	0.04045	< 0.001
Precipitation	0.05363	< 0.001
HH	0.01211	< 0.01
NDWI	0.01065	< 0.05
AET:Precipitation	0.04444	< 0.001
Residues	0.83873	

β-Diversity Partitioning

**Fig. 8:**
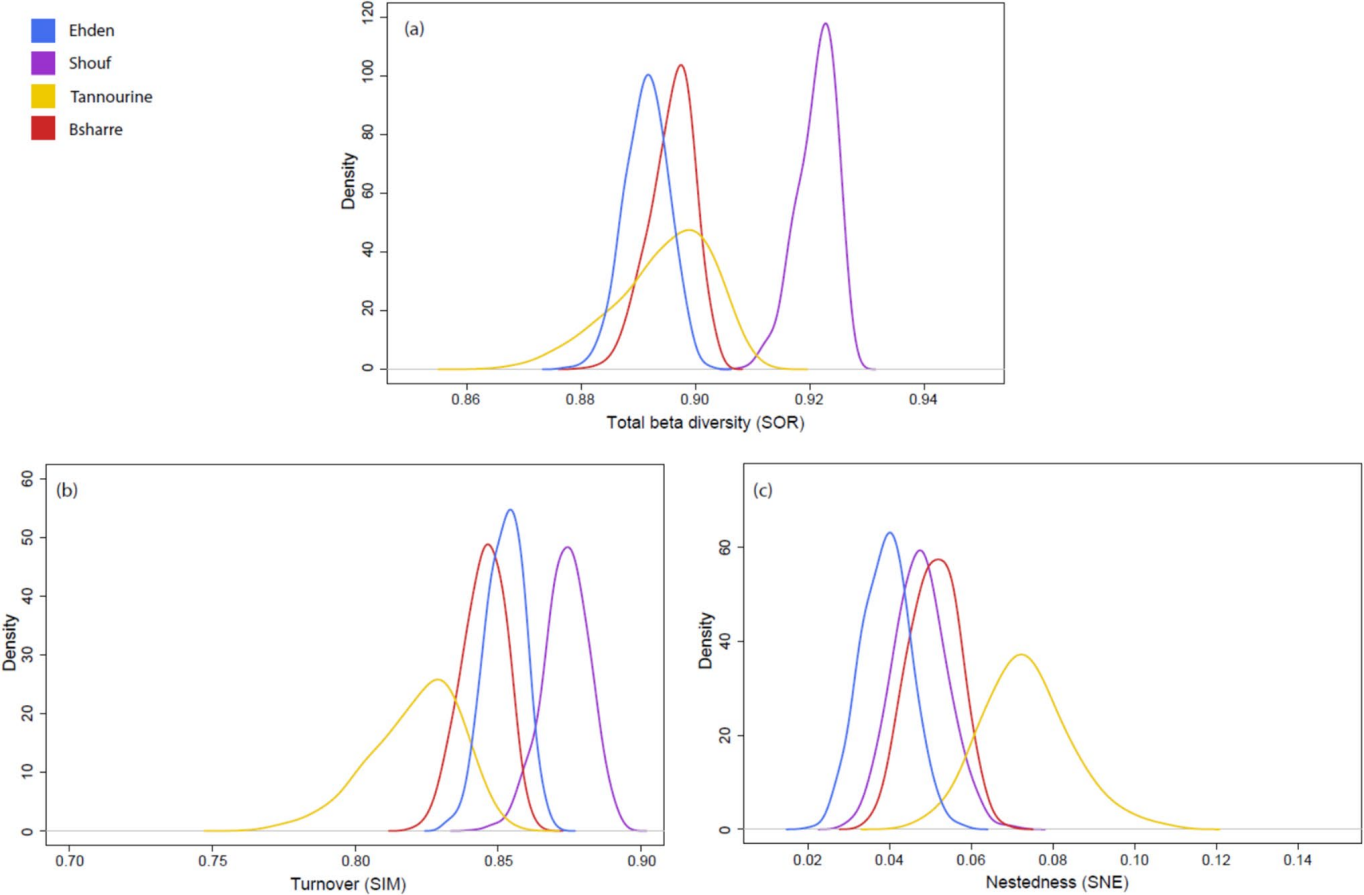
Processes underlying variation in Arcellinida OTU composition across the protected areas. **a**) represents an illustration of total beta diversity (β_SOR_). The beta diversity is partitioned in **b**) turnover, (*i.e*., species replacement between communities due to environmental or spatial differences, β_SIM_) and **c**) nestedness (*i.e*., the extent to which communities are subsets of each other due to non-random species loss, β_SNE_). Kernel density curves were constructed by resampling 20 sites from each area 1000 times and computing the average β_SNE_, β_SIM_ and β_SOR_

Shouf exhibited the highest value of beta diversity, while Ehden had the lowest (Fig. 8c). In general, variance partitioning showed that beta diversity was dominated by turnover. But within all the protected areas, Tannourine presented a wider range of beta diversity among samples (β_SOR_ 0.87–0.91, *P* < 0.001). In line, Tannourine presented a higher value of nestedness (_0.75_, Fig. 8a than Shouf, Ehden, and Bsharre (β_NES_ 0.14, 0.26, and 0.12, respectively; *P* < 0.001). The turnover in Shouf was higher than in the rest of the sites (Fig. 8b), whereas Tannourine had the lowest value of turnover (*P* < 0.001). Ehden showed slightly higher turnover values than Bsharre (Fig. 8b). All of the statistics are included in Supplementary Information Table 5.

## Discussion

### General considerations

Our study was based on a sampling design which allowed examining soil Arcellinida communities accounting for environmental variations due to climate while keeping vegetation type constant. The approach of sampling communities associated to a single plant species to assess the effect of elevation has been applied in the past [40, 89, 90]. However, these studies were based on microscopic observation of rough morphotypes, which cannot reflect the true diversity of Arcellinida given the high amount of cryptic diversity in the group [29, 36, 91]. Moreover, this study is, to our knowledge, the first eDNA survey on Arcellinida in the whole Middle East, where the Levant region represents a well-known diversity hotspot for plants and animals [92, 93]. In line, we retrieved 697 OTUs, which have been built to approximate at best species. While we still lack data to infer if Lebanese cedar forests may also be a hotspot for Arcellinida diversity, these numbers overtake estimations made for invertebrate taxa well-known for their diversity such as Coleoptera and Lepidoptera [94–96]. Indeed, about 500 beetles [97] and 420 moths and butterflies [98] have been reported for the whole country, including all existing biomes. It is therefore not unreasonable to think that Arcellinida alone may be more diverse than animals in a country such as Lebanon.

Infraorder Excentrostoma was, by far, the most represented group. These organisms are abundant in soil, especially in Mediterranean climates like for instance in Greece [99]. This also confirms previous observations based on soil eDNA [29, 55, 58]. While the evolutionary success of Excentrostoma is undisputable, it cannot be ruled out that sequences from this infraorder are preferentially amplified over others. For instance, Hyalospheniformes, a predominantly terrestrial infraorder, is not amplified by this protocol [29]. Interestingly, we obtained a few sequences from infra-orders traditionally present in aquatic environments: seven OTUs of Cylindrothecina, as well as two sequences related to *Netzelia*. These OTUs could represent transitions from aquatic to terrestrial environments.

### Richness Peaks Under Wetter and Warmer Climates

The water-energy balance model states in short that the hotter and wetter the climate, the higher the biodiversity [22]. This theory has been invoked to explain the distribution of testate amoebae diversity in Chile, which peaked in middle latitudes where temperatures are mild and precipitations constant [26]. Among Lebanese cedar forests, Shouf hosted the highest richness in OTUs, followed by Tannourine, Ehden (no statistical difference between the last two), and finally Bsharre (Figs. 5, 6). In line, the environmental variables that were significantly correlated with beta diversity were correlated with both humidity (precipitations, 5%; vegetation water content NDWI 1%), and a combination between humidity and temperature: (actual evapotranspiration AET, 4%). HH, more related to local soil characteristics, explained 1% of the variation (Table 1). The alpha diversity was only impacted by precipitation, therefore showing a higher importance of humidity in the case of species richness and phylogenetic diversity (Table 4). It is important to highlight that the climatic variables have been extracted from raster layers derived from different time periods, although always containing data from recent years. This presents a limitation in this research, but we were unable to obtain data from the same interval of time. The latter have been found to influence the occurrence of pathogenic fungi [100] and may well also have a slight but significant effect on diversity. Nevertheless, Arcellinida richness appears to be mostly correlated with factors that are predicted by the water-energy model. The geographical situation of Shouf is relatively far away of the three other sites (ca. 50 km south) and, given the fact that geographical distance plays a role in Arcellinida distributions [101], some species may have reached their northernmost limit. Such a possibility could also explain partially the higher diversity found in Shouf. Still, even though the effect of moisture and temperature were significant, the variables showed a low R^2^ and therefore a limited explanatory power [102]. Even though our sampling design aimed at reducing acknowledged confounding factors such as vegetation or soil type, other factors, such as biotic ones (competition, predation) may have played a major role in shaping communities.

### Diversity Shifts Between Communities

Communities differed substantially and with a relatively limited overlap between the different protected areas, as illustrated with our NMDS analysis. One exception is Bsharre (high mountain zone), which overlaps to a large extent with the other forests. Its diversity appeared to be to a large extent a subset of communities situated at lower elevations, which suggests that its harsher climatic conditions act as an ecological filter. Of the many species that thrive at lower elevations, only highly stress-tolerant organisms would be capable to persist in Bsharre and would therefore explain the overlap between communities.

Moreover, studies on plant and microbial communities show that productive ecosystems under milder climates host a higher beta diversity, because they allow for greater niche partitioning and species turnover across environmental gradients [100, 103]. Its limited evapotranspiration coefficient and high precipitations (Fig. 4) definitively designate the climate is Shouf as the mildest of all forests studied in this work. Our diversity partitioning analysis showed that beta diversity was mostly driven by species turnover. High turnover is typically found in ecosystems that have many microniches, which promote local adaptation [104]. Protist environmental DNA diversity typically shows high levels of turnover, because of the high number of microniches present in the microbial scale. In our study, the amount of single occurrences of OTUs is rather high (70% of ASVs were exclusive to a single site and 58% were exclusive to one sample) mirroring the situation in other protists and similar studies [58, 105]. This could be due to the heterogeneous nature of cedar litter and the fact that Arcellinida typically have narrow ecological niches [36]. The low dispersal capacity of Arcellinida is also key for maintaining well-defined patterns in the distribution of their diversity [106]. Yet, Tannourine had a higher nestedness than the other forests, even though turnover still was the main driver of its beta diversity. One possibility for this higher nestedness could be attributed to the recent wildfire events, which could have caused local extinctions and subsequent recolonizations. Moreover, Tannourine was a conflict zone during the Lebanese civil war, which brought significant disturbances to biodiversity [48]; recolonization of Arcellinida populations in disturbed sites may take decades [107]

### Possible Consequences of Climate Change on Communities and Effects on Ecosystems

Climate change is already adversely affecting the Mediterranean region, and therefore Lebanon [108, 109]. Indeed, the arid area in Lebanon could increase to over 13% by 2080 [110, 111]. The whole Middle east is undergoing a strong aridification process, with an important decrease in winter precipitations during the last decades [112]. Our results show that humidity and Arcellinida diversity are correlated, therefore the aridification of Lebanon will probably affect the diversity of Arcellinida in the forests. In aquatic environments, global warming has been shown to have decreased testate amoebae diversity [113, 114]; in peatlands, a decrease in functional diversity has also been correlated to drought [115]. Based on these observations and our findings, one can expect a loss of diversity that should affect in priority Bsharre, the driest and coldest environment. A loss of Arcellinida functional diversity, as observed in peatlands, could potentially affect negatively ecosystem functioning [115]. Such phenomenon will be exacerbated by the progressive loss of snow cover in the winter [116], as many Arcellinida are sensitive to frost [117]. Wildfires, which can be expected to become more frequent, will cause a loss of microniches that will impact directly beta diversity through its turnover component [118]. This biodiversity loss would then homogenise diversity, potentially losing redundancy and therefore making ecosystems more vulnerable [119]. Increasing heat, drought and wildfires associated with global climatic change might be putting at risk these unique forests that contributed to the history of humanity for 4,000 years.

Our study on Arcellinida shows that the water-energy balance theory applies to a group of unicellular organisms just as it does for animals and plants, thus expanding the concept to, at least, one group of unicellular organisms. Arcellinida tests show traits in their shells that reveal different adaptive strategies [60]; these traits are well characterized [120]. In vegetation, it has been shown by examining plant traits that warmer and wetter climates host a higher diversity because they permitted a higher number of adaptive strategies (coined the “physiological tolerance hypothesis”; [121]). Observational studies could determine whether functional diversity also decreases in colder and drier sites such as Bsharre, and provide a mechanistic approach of the water energy balance applies to protists.

## Supplementary Information

Below is the link to the electronic supplementary material.

Supplementary Information 1 (CSV 3320 KB)

Supplementary Information 2 (XLSX 9 KB)

Supplementary Information 3 (CSV 13 KB)

Supplementary Information 4 (CSV 0 KB)

Supplementary Information 5 (CSV 6 KB)

Supplementary Information 6 (DOCX 34 KB)

Supplementary Information 7 (ZIP 38 KB)

Supplementary Information 8 (PDF 150 KB)

## Data Availability

The raw data used in this work is available in Zenodo: 10.5281/zenodo.15363229. The bioinformatics pipeline used for the analyses can be found in a compressed folder as Supplementary Information Appendix 3. In the supplementary section we have also provided the environmental data for each sampling site, the sequences of the indexed primers used in this study, the information of the filtered sequences obtained after running DADA2.
