# Supplementary material for: Beneath the Cedars: Exploring the Water-Energy Balance on Arcellinida Biodiversity in Lebanon’s Cedar Forests
Source: Microb Ecol. 2025 Dec 8;89:33. doi: 10.1007/s00248-025-02666-2 (PMC12812088; doi:10.1007/s00248-025-02666-2)
Supplement: Supplementary file 1 [file 248_2025_2666_MOESM8_ESM.pdf]

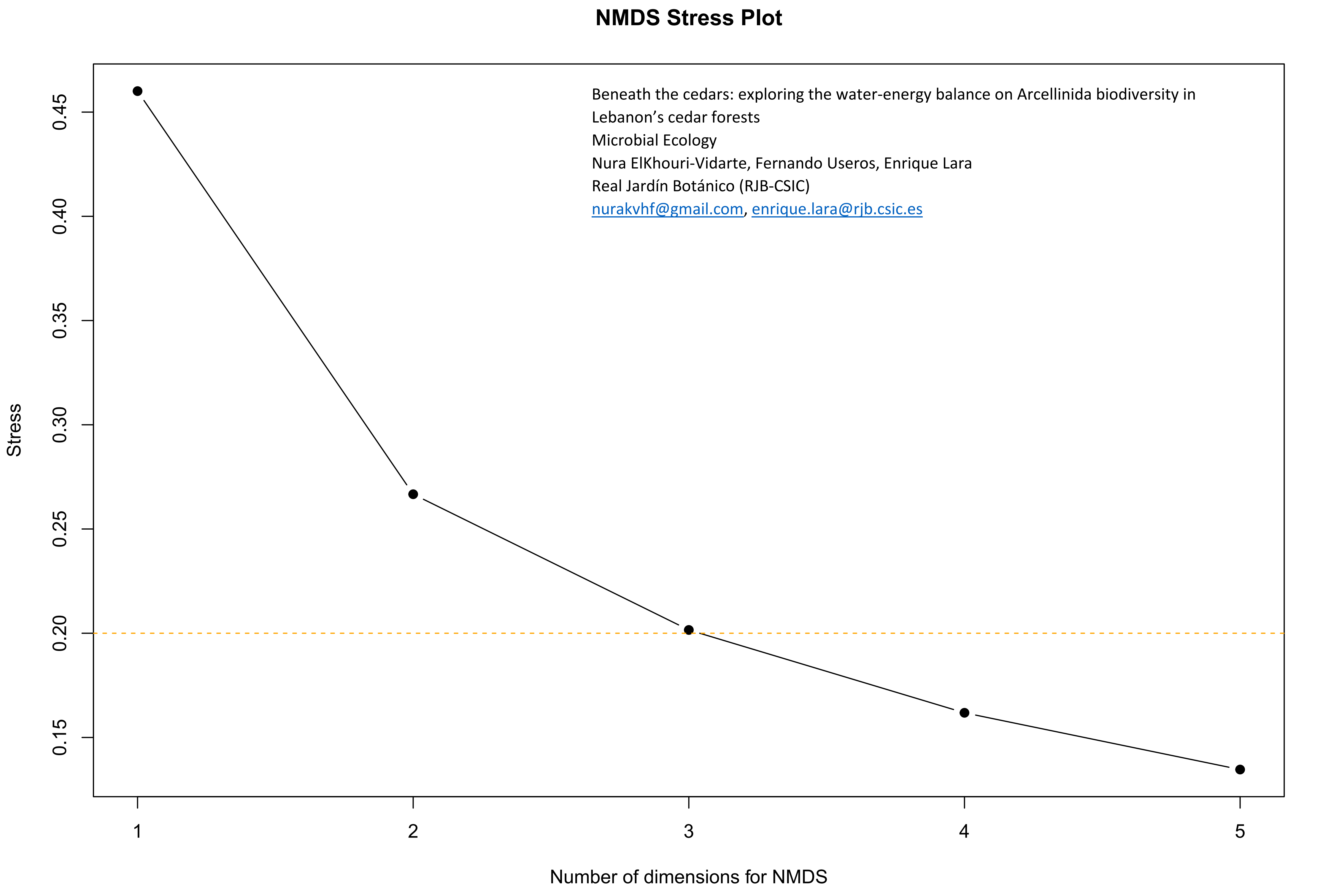
NMDS Stress Plot
Beneath the cedars: exploring the water-energy balance on Arcellinida biodiversity in
Lebanon’s cedar forests
Microbial Ecology
Nura ElKhouri-Vidarte, Fernando Useros, Enrique Lara
Real Jardín Botánico (RJB-CSIC)
nurakvhf@gmail.com, enrique.lara@rjb.csic.es
Stress
0.15
0.20
0.25
0.30
0.35
0.40
0.45
1
2
3
4
5
Number of dimensions for NMDS
