# Supplementary material for: Beneath the Cedars: Exploring the Water-Energy Balance on Arcellinida Biodiversity in Lebanon’s Cedar Forests
Source: Microb Ecol. 2025 Dec 8;89:33. doi: 10.1007/s00248-025-02666-2 (PMC12812088; doi:10.1007/s00248-025-02666-2)
Supplement: Supplementary file 2 [file 248_2025_2666_MOESM6_ESM.docx]

**Supplementary Information**

**Beneath the cedars: exploring the water-energy balance on Arcellinida biodiversity in Lebanon’s cedar forests**

**Microbial Ecology**

**Nura ElKhouri-Vidarte^1^**^, ¶^**, Fernando Useros^1^, Enrique Lara^1^**^, ¶^

**^1^ Real Jardín Botánico (RJB-CSIC), Department of Mycology, Claudio Moyano 1, 28014 Madrid, Spain**

 ¶ Corresponding authors

[nurakvhf@gmail.com](mailto:nurakvhf@gmail.com)**,** [enrique.lara@rjb.csic.es](mailto:enrique.lara@rjb.csic.es)

**Appendix 1**: Extended description of the study region

Bsharre – Horsh Arz el-Rab (HAR, Arabic: أرز الربّ)

This reserve is located on the north-western slopes of the Mount Lebanon area, in the district of Zgharta, North Lebanon. The elevation fluctuates from 1200 m to 2000 m covering over 1000 hectares of which 450 hectares are the protected forest (Ministry of Environment, n.d.). It was declared as protected nature reserve in 1992 (Sattout et al., 2008). The flora is an assemblage of conifers, deciduous and perennial broadleaf trees. Given its diverse topology, HENR has three altitudinal zones: the Supra-Mediterranean (from 1200 m to 1500 m), the Mountainous Mediterranean (from 1500 m to 1900 m) and the Oro-Mediterranean (from 1900 m to 2000 m). HENR belongs to the category Cs according to the Köppen and Geiger climate classification. The annual precipitation in HENR is 1060 mm and the mean relative humidity lies around 60%. The annual temperature is 9.3Cº, while the mean daily maximum is reached in August and is 22.8ºC and the mean minimum is 1.9ºC and is reached in January. This reserved is formed by four forest communities among which we can find the Lebanese Cedar (*Cedrus libani*), the Cilician Fir (*Abies cilicica*), Aleppo Pine (*Pinus halepensis*) and Calabrian Pine (*Pinus brutia*), as well as Palestine Oak (*Quercus calliprinus*) and Aleppo Oak (*Quercus infectoria*) (Abi-Said et al., 2014; *Ehden Climate: Weather Ehden & Temperature by Month*, n.d.; Ramadan Jaradi et al., 2004; Sattout & Caligari, 2011). HENR was declared a nature reserve in 1992, and was recognise as a remnant forest comprising a high biological diversity (Sattout & Caligari, 2011).

Bsharre – Horsh Arz el-Rab (HAR, Arabic: أرز الربّ)

The Cedars of God Reserve (Horsh Arz el-Rab ) is located in the district of Bsharre (North Lebanon), in the western slope of Mount Lebanon. It is a vestige of the historical cedar forest that used to thrive across the country (Naameh, 2020). While cedar forests in Lebanon are an important social and natural heritage of the country, the forest in Bsharre have a particular relevance. Indeed, this forest in already mentioned in the epic of Gilgamesh (Tablets 4-6) and provided wood for the construction of the temple of Jerusalem by King Solomon. 1998, HAR was included in the UNESCO list of World Heritage Sites. The reserve is quite small, it only contains around 350 cedars in 11 hectares (Al Khoury et al., 2021; UNESCO World Heritage Centre, n.d., 1998). Apart from being a national gem and a very touristic place, HAR is also being studied as it is suspected that climate change will severely affect that area. Currently, the forest lies in the oro-mediterranean bioclimatic zone (Colette, 2013). HAR has a weather pattern belonging to the Cs category of the Köppen and Geiger climate classification. The forest’s altitude ranges between 1900 m and 1950 m, its annual precipitation is 950 mm and the average temperature per year is 13ºC, while the mean minimum is reached in January and is 0.3ºC and the mean maximum is reached in August and is 19.7ºC (Al Khoury et al., 2021; *El Arz Climate: Weather El Arz & Temperature by Month*, n.d.)

Maasser El Shouf (Arabic: معاصر الشوف)

Shouf Biosphere Reserve (SBR) reserve is located in the Barouk Mountain range, and is formed by three separated cedar forests: Barouk, Ain Zhalta and Maasser Al-Shouf. It is located in the Shouf District (Mount Lebanon Governorate). This last one was replanted in 1970s as it has suffered a severe fragmentation. The SBR was declared a Nature Reserve in 1996 and is included in the UNESCO-MAN and THE BIOSPHERE Programme since 2005 (Sattout et al., 2008; Sattout & Caligari, 2011)**.** It is the most southernmost limit of Lebanese cedar in the Niha region (Browicz K., 1982). It covers 550 km^2^ and among them 620 hectares are these three patches of pure cedar forest. It represents 5% of the country’s total area (*SBR*, n.d.). The SBR ranges between 1200 m and 1980 m, but the Maasser has an altitude ranging from 1600 m and 1750 m. Maasser al-Shouf covers only 16 hectares but contains one of the oldest cedar trees in the country (*Shouf Biosphere Reserve*, n.d.). Maasser Al-Shouf is classified as Csa according to the Köppen and Geiger climate classification average annual precipitation is around 600 mm, the average temperature is 14.2ºC, the average humidity is 64.25%, the maximum temperature is reached in August and is 28.9ºC, while the minimum temperature is reached in January and is 0.1ºC (Hani et al., 2017; *Maasser Ech Chouf Climate: Weather Maasser Ech Chouf & Temperature by Month*, n.d.; Sattout & Caligari, 2011)**.**

Tannourine (Arabic: تنورين) Cedar Forest Nature Reserve (TCFNR)

The TCFNR was declared in 1999 by the Ministry of Environment and is one of the largest Cedar Reserves in Lebanon. It is located in the Batroun District (North Lebanon), the reserve extends on the south-western and north-eastern slopes and occupies around 1.5 km^2^, around 625 hectares (Al Khoury et al., 2021; Bassil et al., 2018; Sattout, 2007)**.** It is a very rich reserve as among the more than two hundred species inventoried in it, twenty-five are endemic and represent more than the 50% of the endemic plants in the country (Ghattas et al., 2003). The soils at TCFNR are calcareous, sandy and volcanic. It includes the largest Cedar forest in Lebanon, Hadath El-Jebbeh (Ghattas et al., 2003; Sattout & Nemer, 2008)**.** The altitude ranges between 1200 m and 1900 m. It is considered one of the older remnant patches of cedar forest, the *C. libani* being the dominant species with a relative density of 90% (Mitri & El Hajj, 2007). Apart from cedars, at TCNFR we can find other species as *Juniperus excelsior*, *Juniperus oxycedrus*, *Cupressus sempevirens*, *Quercus calliprinos*, *Piris syriaca* or *Prunus ursina* (Sattout, 2007).

The TCFNR is classified as Cs according to the Köppen and Geiger climate classification. The rainfall at TCNFR is around 553 mm per year, with a mean humidity of 64.1%. January is the coldest month, with the minimum temperature reached at -1.2ºC and August is the warmest one, reaching 25.7ºC, the maximum temperature. The average temperature is 12ºC. (*Tannourine El Faouqa Climate: Weather Tannourine El Faouqa & Temperature by Month*, n.d.)

**Appendix 2**: Raw data obtained by Illumina NextSeq sequencing.

**Appendix 3**: Whole pipeline to do the demultiplexing, cleaning, taxonomic assignation and to obtain the results in this study.

**Appendix 4:** Stress curve of the NMDS, showing the stress value for each value of k, from 1 to 5.

Tables

**Table 1:** Environmental data for each sampling site. It shows for each sample the combination of primers used in the column “variable”, the Park where it was collected, the coordinates X and Y, the elevation, number of ASVs and four climatic variables extracted from the raster layers: HH (PALSAR_HH), norm_perceqalized difference water index (NDWI), precipitation and actual evapotranspiration (AET). The unused combinations of indexed primers that have been used to determine thresholds to clean up probable tag-jumps are listed as “Unused”.

**Table 2**: Name and sequence of the primers used in this study. The first eight nucleotides correspond to the index, and the rest to the primer sequence.

| **Name** | **Sequence** |
| --- | --- |
| **ARCOIR-a** | **CAGAGACG**CCACYNGAATGWGCTARAATACC |
| **ARCOIR-b** | **CAGATGAC**CCACYNGAATGWGCTARAATACC |
| **ARCOIR-c** | **CAGTATGC**CCACYNGAATGWGCTARAATACC |
| **ARCOIR-d** | **CATAGTAT**CCACYNGAATGWGCTARAATACC |
| **ARCOIR-e** | **CATGTGCT**CCACYNGAATGWGCTARAATACC |
| **ARCOIR-f** | **CGAGAGAC**CCACYNGAATGWGCTARAATACC |
| **ARCOIR-g** | **CGAGTACG**CCACYNGAATGWGCTARAATACC |
| **ARCOIR-h** | **CGATGTAG**CCACYNGAATGWGCTARAATACC |
| **ARCOIR-i** | **CGTATAGC**CCACYNGAATGWGCTARAATACC |
| **ARCOIR-j** | **CGTGATGT**CCACYNGAATGWGCTARAATACC |
| **ARCOIR-k** | **GAGATAGT**CCACYNGAATGWGCTARAATACC |
| **ARCOIR-l** | **GAGTGTCT**CCACYNGAATGWGCTARAATACC |
| **ARCOIR-m** | **GCATATAC**CCACYNGAATGWGCTARAATACC |
| **ARCOIR-n** | **GCATGACG**CCACYNGAATGWGCTARAATACC |
| **ARCOIR-o** | **GCGAGTAG**CCACYNGAATGWGCTARAATACC |
| **ARCOIR-p** | **GCTATGAT**CCACYNGAATGWGCTARAATACC |
| **ARCOIR-q** | **GCTGATCG**CCACYNGAATGWGCTARAATACC |
| **ARCOIR-r** | **TAGTAGAG**CCACYNGAATGWGCTARAATACC |
| **ARCOIR-s** | **TATGATAC**CCACYNGAATGWGCTARAATACC |
| **ARCOIR-t** | **TCAGTAGT**CCACYNGAATGWGCTARAATACC |
| **ARCOIR-u** | **TCATAGCT**CCACYNGAATGWGCTARAATACC |
| **ARCOIR-v** | **TCGATGCG**CCACYNGAATGWGCTARAATACC |
| **ARCOIR-w** | **TCTAGTGC**CCACYNGAATGWGCTARAATACC |
| **ARCOIR-x** | **TGATGAGC**CCACYNGAATGWGCTARAATACC |
| **ARCOIR-y** | **TGTAGACT**CCACYNGAATGWGCTARAATACC |
| **ARCOIR-z** | **TGTGTGAG**CCACYNGAATGWGCTARAATACC |
| **LCO-L** | **CGACTCAT**GGTCAACAAATCATAAAGATATTGG |
| **LCO-M** | **CGAGACGC**GGTCAACAAATCATAAAGATATTGG |
| **LCO-N** | **CGAGCACA**GGTCAACAAATCATAAAGATATTGG |
| **LCO-O** | **CGTATCGA**GGTCAACAAATCATAAAGATATTGG |
| **LCO-P** | **TAGACAGT**GGTCAACAAATCATAAAGATATTGG |
| **LCO-Q** | **TAGCACGA**GGTCAACAAATCATAAAGATATTGG |
| **LCO-R** | **TATGACAC**GGTCAACAAATCATAAAGATATTGG |
| **LCO-S** | **TATGTAGA**GGTCAACAAATCATAAAGATATTGG |

**Table 3**: Statistic information of the filtered sequences after the DADA2 cleaning step.

**Table 4**: Information about the number of Arcellinida reads and percentage of Arcellinida reads among the total results.

**Table 5:** Statistics of the total and partitioned beta diversity among sites. Values correspond to Dunn’s post-hoc test results after the Kruskal-Wallis test. Beta diversity (β_SOR_) is partitioned into turnover (β_SIM_) and nestedness (β_NES_). The Z-values represent the standardized differences between site pairs, and adjusted P-values indicate statistical significance after multiple comparisons correction. Significant differences (P < 0.05) suggest variation in beta diversity components between sites.

| **Comparison** | **Nestedness (β_NES_)** | **P (adjusted)** | **Turnover (β_SIM_)** | **P (adjusted)** | **Beta Diversity (β_SOR_)** | **P (adjusted)** |
| --- | --- | --- | --- | --- | --- | --- |
| **Bsharre - Ehden** | 19.25 | 4.42×10⁻⁸² | -16.92 | 8.69×10⁻⁶⁴ | 12.57 | 9.40×10⁻³⁶ |
| **Bsharre - Shouf** | -19.29 | 1.75×10⁻⁸² | -36.81 | 3.84×10⁻²⁹⁶ | -41.06 | 0 |
| **Ehden - Shouf** | -38.54 | 0 | -19.88 | 1.68×10⁻⁸⁷ | -53.63 | 0 |
| **Bsharre - Tannourine** | -38.74 | 0 | 20.81 | 9.74×10⁻⁹⁶ | -19.59 | 5.07×10⁻⁸⁵ |
| **Ehden - Tannourine** | -57.98 | 0 | 37.73 | 0 | -32.16 | 1.67×10⁻²²⁶ |
| **Shouf - Tannourine** | -19.44 | 9.45×10⁻⁸⁴ | 57.62 | 0 | 21.47 | 8.45×10⁻¹⁰² |
