# Supplementary figures and images for: Beneath the Cedars: Exploring the Water-Energy Balance on Arcellinida Biodiversity in Lebanon’s Cedar Forests
Source: Microb Ecol. 2025 Dec 8;89(1):33. doi: 10.1007/s00248-025-02666-2 (PMC12812088; doi:10.1007/s00248-025-02666-2)

## NMDS Stress Plot

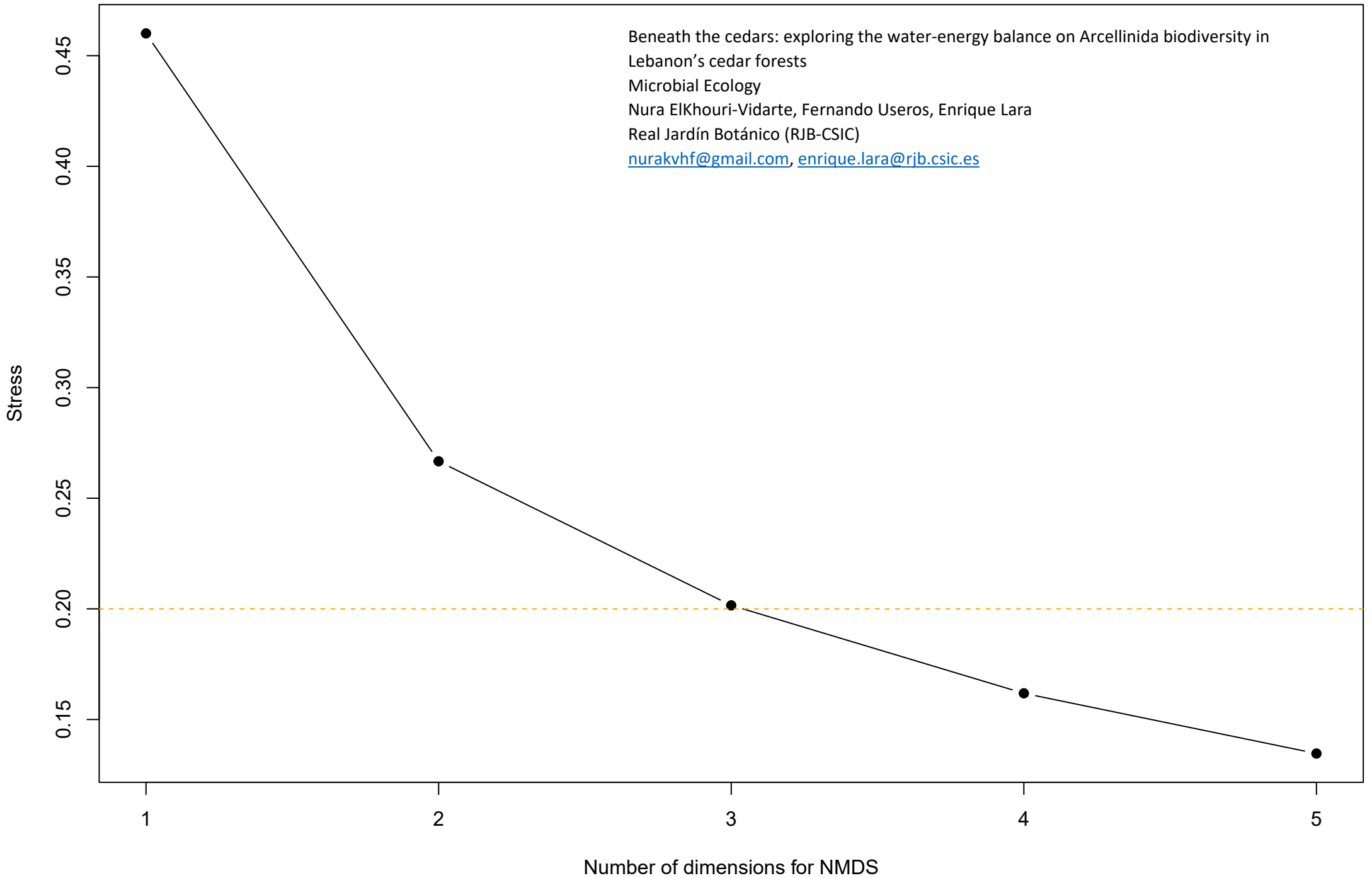

Supplement: Supplementary file 8 — Supplementary Information 8 (PDF 150 KB) [file 248_2025_2666_MOESM8_ESM.pdf]
